# An Extremely Rare Cause of an Obstructive Jaundice in Adults: Limited Langerhans Cell Histiocytosis of the Extrahepatic Bile Duct

**DOI:** 10.1159/000521972

**Published:** 2022-02-07

**Authors:** Saeed Aldarwish, Clemens Schafmayer, Andreas Erbersdobler, Sebastian Hinz

**Affiliations:** ^a^Department of General-, Visceral-, Thoracic-, Vascular- and Transplant Surgery, University Hospital Rostock, Rostock, Germany; ^b^Department of Pathology, University Hospital Rostock, Rostock, Germany

**Keywords:** Langerhans cell histiocytosis in adults, Extrahepatic bile duct, Obstructive jaundice, Limited histiocytosis, Cholangiocarcinoma

## Abstract

Langerhans cell histiocytosis (LCH) is a rare group of idiopathic disorders (previously termed “histiocytosis X”) which is characterized by the presence of cells with characteristics similar to bone marrow-derived Langerhans cells which infiltrate various tissues and organs. Like Langerhans cells located in the skin, they express histiocytic markers such as S100, CD1a, and CD68 and contain Birbeck granules, which are rod-shaped intracytoplasmic organelles best demonstrated in electron microscopy. LCH primarily affects the skeleton, but lung, skin, liver, and lymph node involvement may occur alike. Hepatic involvement is well recognized in children, with sclerosing cholangitis occurring in 10–15% of those with multisystemic involvement, whereas LCH confined to the liver appears to be very unusual in adults. Up to date, only one case of a solitary LCH affliction of the extrahepatic bile duct lacking liver involvement in adulthood has been reported on in the literature. We here report on a 60-year-old male patient with classical indolent progressive obstructive jaundice. The diagnostic workup revealed a tumorous lesion in the middle third of the common hepatic bile duct, initially being highly suspicious of an extrahepatic cholangiocarcinoma. CT scans further suspected an infiltration of the right portal vein, implicating a potentially extensive tumor growth, but a preoperative histological confirmation was not feasible. The serum tumor marker CA19-9 was 48 U/mL. The patient then underwent an explorative laparotomy with a pylorus preserving pancreaticoduodenectomy, as all frozen section tissue specimens revealed no tumor infiltration. The final result of the histopathological examination revealed an isolated LCH in the extrahepatic bile duct with a consecutive secondary sclerosing cholangitis. To complete the tumor staging, a thorax CT scan was performed and a generalized histiocytosis was ruled out, hence confirming the localized character of the disease. To the best of our knowledge and after a comprehensive literature review, we report on the second case globally, which describes a primary LCH limited to the extrahepatic bile duct in adulthood. A generalized sclerosing cholangitis in the liver was ruled out by radiological imaging. Preoperative histological affirmation of such findings is very confined due to the complexity and hence can only be diagnosed in the postoperative specimen. However, patients with nondisseminated sole findings, usually report a good prognostic outcome after surgical resection despite the paucity of corresponding data.

## Introduction

The working group of the Histiocyte Society has divided histiocytic disorders into three subgroups: (1) dendritic cell histiocytosis, (2) macrophage-related disorders, and (3) malignant histiocytosis [1]. Langerhans cell histiocytosis (LCH) comes under subgroup (1) being a rare group of idiopathic disorders characterized by the presence of cells with properties similar to bone marrow-derived Langerhans cells. These are juxtaposed against a backdrop of hematopoietic cells, including T-cells, macrophages, and eosinophils. LCH is clinically most often characterized by single or multiple osteolytic bone lesions or skin rashes exhibiting an infiltration of histiocytes with “bean-shaped” nuclei on biopsy, with or without histiocytic infiltration of extra skeletal tissues [2]. Any organ tissue of the human body can be affected, but those more frequently involved are the skeleton (80% of cases), skin (33%), and pituitary gland (25%). Less commonly involved are the liver (15%), spleen (15%), the hematopoietic system (15%), the lungs (15%), lymph nodes (5–10%), and the central nervous system (2–4%) [3]. LCH can affect patients from the neonatal period to adulthood, although it appears to be more common in children aged 0–15 years (reportedly approximately 4 cases per 1 million population) with a 2:1 female preponderance [4]. The clinical course may vary from a self-limiting disease to a rapidly progressive and possibly fatal extend. Between 30% and 40% of patients may develop permanent adverse sequelae. On the one end, the clinical spectrum includes an acute, fulminant, disseminated disease formerly called “Letterer-Siwe”-disease, on the other end, solitary or few, indolent and chronic lesions of the bone or other organs, called eosinophilic granulomas, can be the only affection of the patients with hence a mild clinical depiction. The intermediate clinical presentation, formerly called “Hand-Schüller-Christian”-disease, is characterized by a multifocal, chronic involvement, and typically unveils as the triad of diabetes insipidus, proptosis, and lytic bone lesions. A congenital, self-healing form, formerly called “Hashimoto-Pritzker”-disease, has also been described in the literature [5]. Though, summarizing, the present WHO classification only differentiates between the local and disseminated disease. Since 2016, the revised classification of histiocytosis and neoplasms of the macrophage-dendritic cell lineages is broadly applied [6]. LCH may produce a picture of sclerosing cholangitis in both, children [5] and adults [7], possibly as a result of the bile duct infiltration by the Langerhans cell granulomatous tissue. Liver involvement in LC usually is regarded as a late-onset complication; however, cholestasis can be the first clinical symptom and presentation [5]. In their data, the French Langerhans Cell Histiocytosis Study Group described LCH affection of the liver tissue in 10.1% of patients during the first illness episode, which can then upsurge to 14.4% throughout the course of disease, with a sclerosing cholangitis accounting for 1.3% of cases [8].

## Case Presentation

**Fig. 1:**
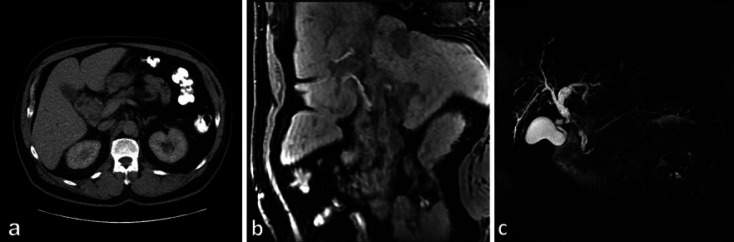
Radiological workup. **a** A computer tomography of the abdomen, axial view. **b** T1 weighted magnetic resonance of the abdomen, coronal view. **c** Magnetic resonance cholangiopancreaticography. A space-occupying mass measures about 31 × 35 × 51 mm arising from the middle third of the common hepatic duct with a suspected infiltration in the porta hepatis and projection in the head of pancreas.

**Fig. 2:**
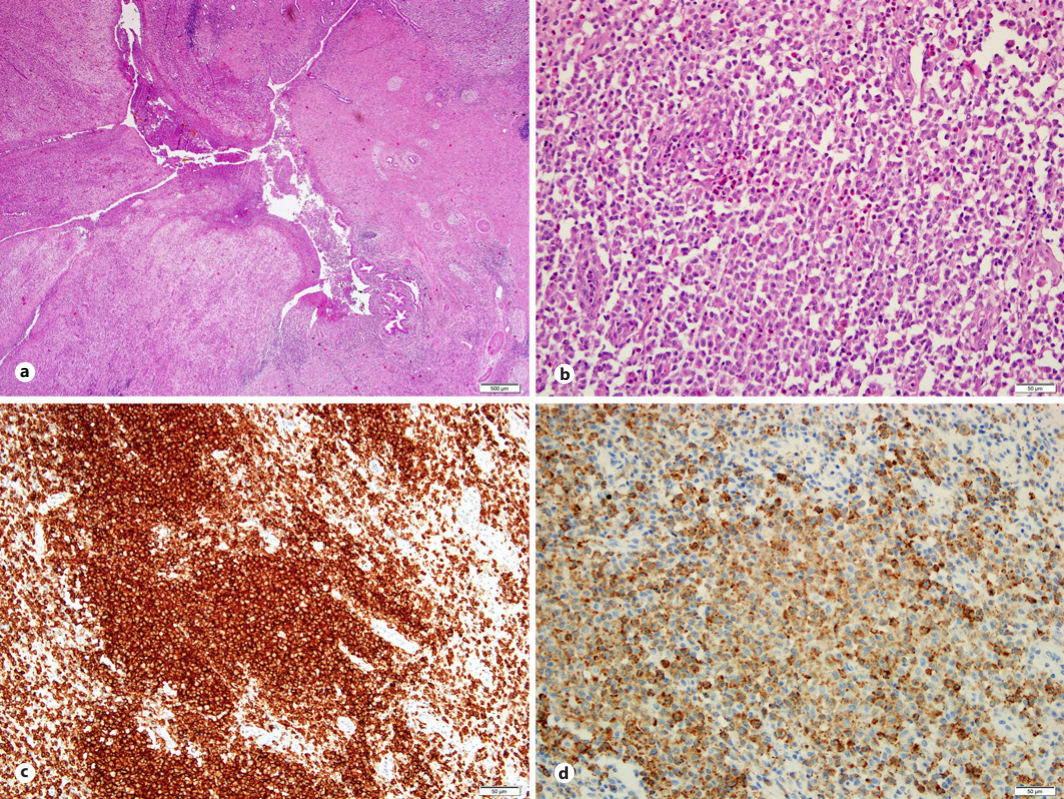
Histopathological findings. Tumorous thickened intrapancreatic bile duct with (×20 magnified, 500 µm) (**a**), Clusters of Langerhans cells with intermediate size nuclei, nuclear grooves, and irregular nuclear contours admixed with eosinophils and plasma cells (×200 magnified, 50 µm) (**b**). **c, d** The majority of Langerhans cells co-express CD1a (**c**) and Langerin (CD207) (**d**) (×200 magnified, 50 µm).

A 60-year-old male patient was referred to our clinic with the suspicion of an extrahepatic cholangiocarcinoma in the distal part of the common bile duct. The patient reported of symptoms such as painless obstructive jaundice with a gradual onset over 3 weeks, weight loss, or other accompanying symptoms were negated. As secondary diagnosis obesity (grade II) and mild arterial hypertension were recorded. A contrast-enhanced CT scan of the abdomen and a liver MRI then revealed a tumorous lesion in the middle third of the common hepatic duct measuring 31 × 35 × 51 mm with a consecutive dilated proximal biliary tree. Further, an infiltration of the right portal vein was diagnosed (shown in Fig. 1). In the initial blood sampling, the total serum bilirubin was elevated to 92 μmol/L (87 μmol/L direct bilirubin). There was no clinical or paraclinical indication to an accompanying cholangitis or pancreatitis. As an incipient bridging treatment procedure to prevent further stenosis of the bile duct, a biliary stent was inserted by endoscopic retrograde cholangiopancreatography. An endoscopic transpapillary tissue biopsy for histopathological examination of the bile duct was not feasible due to the unavailability of this procedural expertise in our institution. To the best of our knowledge, there are no other potential biopsy techniques indicated and feasible in the case of suspecting an extrahepatic cholangiocarcinoma. The preoperative tumor marker CA19-9 was slightly elevated. With the now suspected condition of an extrahepatic cholangiocarcinoma and completion of the preoperative diagnostic workup, the patient was surgically explored. Intraoperatively, the tumor was firm but not fixed, located in the middle to distal part of the extrahepatic common bile duct, but in contradiction to the preoperative radiological work-up, lacking infiltration of the adjacent structures. All of the frozen section's biopsies failed to detect a malignancy. The surgical resection was performed in terms of a pylorus preserving pancreaticoduodenectomy. The final postoperative histopathological examination then unexpectedly revealed a primary LCH of the common bile duct with an accompanying erosional cholangitis. The immunohistochemical staining presented expression of Langerin, CD1a and S100 in the subepithelial cells, which conclusively confirmed the diagnosis of a LCH of the common bile duct (shown in Fig. 2). The postoperative tumor screening with a chest CT scan was negative for further LCH lesions, henceforth, the condition was addressed as a primary limited LCH to the extrahepatic bile duct.

The patient was discharged after 20 days in good clinical reconstitution. Fifteen months after the operation the patient still reports well-being without any further specific therapy. The patient underwent MRCP and routine liver biopsy 12 months after the operation showing no signs of an intrahepatic LCH.

## Discussion

LCH is a rare disease characterized by clonal proliferation of pathologic cells that have the characteristics of Langerhans cells [9]. Langerhans cells are bone marrow-derived dendritic cells that normally reside in the skin and lymph nodes [10]. The final etiology of LCH still remains unknown. The clinical presentation of LCH is heterogeneous and can involve single or multiple organs. Patients with LCH show an infiltration of abnormal cells, which eventually results in manifest organ dysfunction. Depending on the extent of organ involvement, LCH can range from benign disease to a multisystem life-threatening condition [11]. It remains unknown whether LCH is of reactive or neoplastic source. Clonal histiocytes have been detected in most patients with LCH, which hence insinuates a neoplastic disorder [12]. However, the alternative hypothesis sees LCH as a reaction against an infective agent that results in an immune dysregulation [12]. LCH generally is classified as either a single or multisystem condition. Single-system LCH is characterized by involvement of a single site (unifocal bone, skin, or lymph node) or involvement of multiple sites within the same organ system (multifocal bone or multiple lymph nodes). Multisystem LCH is characterized by involvement of two or more differing organ systems at diagnosis, with or without organ dysfunction [11]. Involvement of the liver, lungs, spleen, or hematopoietic system is often associated with a poor prognosis and high mortality rate [11]. Liver involvement is usually seen in the disseminated form of LCH and clinically presents with a hepatomegaly as the cause of histiocyte infiltration into the hepatic sinusoids and portal tracts. Jaundice, liver dysfunction, and ascites are the most common clinical presentations. Hepatic involvement is also well recognized, especially in children with sclerosing cholangitis occurring in 10–15% of those with multisystemic involvement [5, 13], whereas LCH confined to the liver appears to be very unusual [14, 15]. Kaplan et al. [16] reported on 9 cases of hepatobiliary LCH and located another 85 acceptable cases in the literature. The patients' ages ranged from 7 days to 62 years (median 18 months), with a 2:1 female preponderance [16]. Biliary involvement secondary to LCH can also occur in the disseminated form of the disease. In these cases, the prognosis is uniformly poor [5, 17]. Fortunately, biliary involvement, which can manifest as an infiltration of intrahepatic and extrahepatic bile ducts, is a rare occurrence. Jaundice is considered to be a bad prognostic sign, as biliary involvement is usually progressive. Langerhans cell infiltration of the bile duct can exhibit itself with a progressive destruction and secondary sclerosing cholangitis, ultimately leading to secondary biliary cirrhosis [18]. In fact, some authors have suggested that some cases of primary sclerosing cholangitis are related to biliary involvement in patients with LCH. In advanced cases, differentiating these two conditions would be difficult. However, in contrast to patients with primary sclerosing cholangitis, the involvement of extrahepatic ducts is rarely seen in patients with LCH [7].

The therapeutic modalities vary from an observational up to a radical surgical sanitation and multimodal radiochemotherapy. There is no standardized treatment of LCH in general, which has been established in larger randomized trials. Individualized treatment plans depend upon the individual patient, the extent and areas of involvement. There are several reports of successful treatment with a combinational therapy of vinblastine and steroids [19], while other patients have benefited from limited surgery, other chemotherapy agents such as 2-chlorodeoxyadenosine or methotrexate, and even low-dose external beam irradiation [20]. Some patients may have limited involvement, which does not progress to other areas, and may not need systemic treatment [21]. Therefore, treatment of choice is highly individualized to each clinical presentation of LCH considering the involved organs, extent of disease, age of the patient and the accompanying comorbidities. Until now, globally, only one case with a limited LCH to the extrahepatic bile duct in an adult is reported in the literature [22]. We are reporting the second case of a primary confined LCH in the extrahepatic bile duct in an adult male but with the absence of an accompanying sclerosing cholangitis [14, 22].

## Conclusion

Liver and bile duct involvement is usually observed in the disseminated form of LCH. The provisional diagnosis of LCH should be considered when the patient shows other associating clinical and radiological signs. Generally, LCH is a challenging diagnosis for both clinicians and radiologists. In most cases, preoperative histological specimen retrieval is particularly challenging, and hence, most patients are operated on without a preoperatively proven histology. In our presented incident, there was no accompanying sclerosing cholangitis in contrast to other published cases. Due to the extreme scarcity of cases of LCH in the biliary tract and paucity of corresponding studies, there is no generally recommended management of either a treatment of choice or a follow-up strategy. Valid prognostic data are also deficient. However, routine liver ultrasound or MRI examinations of the liver should be performed as routine follow-up.

## Statement of Ethics

The approval of the Ethics Committee of the University Medical Facility of Rostock was obtained (Registration number: A 2021-0195). All authors state that subjects have given their written informed consent to publish their case. A written informed consent was obtained from participants and from the patient for publication of the details of their medical case and accompanying images.

## Conflict of Interest Statement

The authors have no conflict of interest to declare.

## Funding Sources

No funding was received.

## Author Contributions

S.A. reviewed the literature, participated in the design of the study, data analysis, and paper writing. S.H. and C.S. participated in the design of the study and critically evaluated the manuscript. A.E. prepared the histopathological figures and corresponding data of the case. All authors read and approved the final manuscript.

## Data Availability Statement

All data generated or analyzed during this study are included in this article. Further enquiries can be directed to the corresponding author.
